# Observations on the Extraction of Teeth

**Published:** 1802-08-01

**Authors:** 

**Affiliations:** Bond Street


### *Obfervations on the Extraction of Teeth; communicated by Mr.* FOWLER, *of Bond Street.*

IN the Medical and Phyfical Journal of laft year, I was a little furprifed to find that a profeffional gentleman fhould voluntarily come forward, to recommend an inftrument for the extraction of teeth in a *perpendicular* direction; but now I am the more furprifed, to find the fame gentleman abandoning his recommendation, (when requefted to favour the public with a drawing of it) and fubftituting a production of his own in its ftead, which by no means anfwers either Mr. Cuftance's requeft, or the general expectation.

I have not the pleafure of being acquainted with Mr. Cuftance, of courfe, cannot be fufpected of any prefentiment in his favour; but when I read his requeft, accompanied with remarks, I concluded they were fuch as would guide a man of fcience in the profecution of his profeffion. Every Anatomift knows that the large MOLARES (which I take for granted are the principal objects of attention) have their prongs fo completely enveloped in bone, and thefe diverfified in fuch directions, as to make *perpendicular* extraction impoffible, (except in fome favourable cafes,) without fracturing the whole of the furrounding alveola, or the neck of the tooth; this I believe to be generally acknowledged: Nor is this all, the adjacent tooth muft fuffer by depreffion proportionably to the power neceffarily exerted to raife its offending neighbour.

With refpect to Mr. Witford's, or Mr. Simfon's patent inftruments, I confider them as mafter-pieces of ingenuity; infomuch, that if either had been rightly formed, they moft probably could have produced an inftrument which would have needed no partial eulogium; nor have rendered themfelves liable to the mortification of a rival in the ODONTAGRA.

Whoever has attended to the anatomical fituation of the large MOLARES (in a general way) will be convinced, that in order to extract them a fracture muft be produced fomewhere; and that the external part of the alveola appears more favourable for fuch neceffary violence than any other; if fo, the lateral motion which the common key inftrument occafions, muft be *(in judicious hands)* the beft mode of extraction. If the ODONTAGRA has any advantage attending it, it is that of a fpring to fix the claw upon the tooth; but the external projection of this is a confiderable objection, efpecially as the preffure of a finger upon the common claw will anfwer every fuch purpofe.

I believe no furgical operations are attended with more uncertainty, or more mortification, than the extraction of teeth: It is not then to be wondered at, that men of reputation in that refpectable department, fhould confign to others fo trifling an object, fraught with much rifk. However, from the intercourfe I have had with other Dentifts, I can affure Mr. Reece that he will not have it long to lament, that this practice is not more in the fchool of Hippocrates, as I believe moft Dentifts in town plainly fee the propriety of training up their pupils in Anatomical and Medical ftudies, many of whom are now in practice, and, no doubt, in the courfe of a few years, will have the preference.

It would be a happy circumftance both for patient and practitioner, if Mr. Reece's defcriptive ftate of the prongs or ftumps was correct: Whenever the alveolar procefs is "abforbed," (which is comparatively feldom) there will never be any need of fo formidable an inftrument as the ODONTAGRA; for the point of a probe will be fufficient to elevate them with eafe.

We have every reafon to lament with Mr. Reece, that the extracting of teeth is not in more judicious hands; but I fear from his cafe of the Monmouthfhire lady, that her furviving friends will be very loath to prefer the Surgeon's knowledge to the practice of even "ignorant people:" For it fhould feem, that if a common tooth-drawer had been fent for, he would have have taken out the tooth, and all would have been well; whereas, the little inflammation of the gums (which generally accompanies tooth-ach) was increafed by delay, till at length the patient loft her life. I hope thofe "medical attendants who are very defervedly efteemed gentlemen of great profeffional judgment and fkill," will not hefitate in future to extract a tooth on account of an inflamed gum.

In general, whenever inflammation arifes from difeafed teeth, the fooner they are removed, the fooner will the inflammation fubfide. During twenty years practice, I have never feen one bad effect arifing from inflammation of the gums, when I could prevail upon my patients to fubmit to the operation; but many very bad confequences have been occafioned by delay, and one fimilar to the foregoing terminated fatally, an account of which I have already publifhed in a former Number of your ufeful Mifcellany.

*March* 10, 1802.

